# Women’s experiences in a community-based screen-and-treat cervical cancer prevention program in rural Malawi: a qualitative study

**DOI:** 10.1186/s12885-021-08109-8

**Published:** 2021-04-22

**Authors:** Fan Lee, Agatha Bula, John Chapola, Clement Mapanje, Billy Phiri, Nenani Kamtuwange, Mercy Tsidya, Jennifer Tang, Lameck Chinula

**Affiliations:** 1 University of North Carolina (UNC) Department of Obstetrics and Gynecology, Chapel Hill, USA; 2 UNC-Project Malawi, Lilongwe, Malawi; 3 Kamuzu Central Hospital, Lilongwe, Malawi

**Keywords:** Malawi, Cervical cancer screening, VIA, Thermocoagulation, Acceptability, Barriers, HPV self-collection

## Abstract

**Background:**

Malawi has the world’s highest cervical cancer incidence and mortality due to high rate of HIV coupled with inadequate screening and treatment services. The country’s cervical cancer control program uses visual inspection with acetic acid (VIA) and cryotherapy, but screening is largely limited by poor access to facilities, high cost of cryotherapy gas, and high loss-to-follow-up. To overcome these limitations, we implemented a community-based screen-and-treat pilot program with VIA and thermocoagulation. Through a qualitative study, we explore the experiences of women who underwent this community-based pilot screening program.

**Methods:**

We implemented our pilot program in rural Malawi and conducted an exploratory qualitative sub-study. We conducted in-depth interviews with women who were treated with thermocoagulation during the program. We used semi-structured interviews to explore screen-and-treat experience, acceptability of the program and attitudes towards self-sampling for HPV testing as an alternative screening method. Content analysis was conducted using NVIVO v12.

**Results:**

Between July – August 2017, 408 participants eligible for screening underwent VIA screening. Thirty participants had VIA positive results, of whom 28 underwent same day thermocoagulation. We interviewed 17 of the 28 women who received thermocoagulation. Thematic saturation was reached at 17 interviews. All participants reported an overall positive experience with the community-based screen-and-treat program. Common themes were appreciation for bringing screening directly to their villages, surprise at the lack of discomfort, and the benefits of access to same day treatment immediately following abnormal screening. Negative experiences were rare and included discomfort during speculum exam, long duration of screening and challenges with complying with postprocedural abstinence. Most participants felt that utilizing self-collected HPV testing could be acceptable for screening in their community.

**Conclusions:**

Our exploratory qualitative sub-study demonstrated that the community-based screen-and-treat with VIA and thermocoagulation was widely accepted. Participants valued the accessible, timely, and painless thermocoagulation treatment and reported minimal side effects. Future considerations for reaching rural women can include community-based follow-up, cervical cancer education for male partners and self-sampling for HPV testing.

**Supplementary Information:**

The online version contains supplementary material available at 10.1186/s12885-021-08109-8.

## Background

Cervical cancer is preventable through early detection and treatment of precancerous lesions. However, cervical cancer remains the 4th most common cancer in women worldwide and the leading cause of cancer deaths in Sub Saharan Africa (SSA) [1, 2]. In SSA and other low-and-middle-income countries (LMICs), high cervical cancer incidence and mortality is related to high rates of HIV [3, 4] coupled with inadequate screening and treatment services for precancerous lesions [5]. Malawi has the world’s highest age-standardized incidence rate of cervical cancer (72.9 per 100,000 persons) and the highest recorded mortality at 54.5 per 100,000 persons [1]. The prevalence of HIV is also high, at about 8.8% in adults age 15–49 years old [6]. Effective cervical cancer prevention strategies require equipment, trained health care workers, ease of access to health services and community acceptability of screening and treatment, all of which have been cited as barriers to adequate screening coverage in LMICs [7, 8]. To overcome some of these barriers, efforts have focused on single-visit screen-and-treat strategies that utilize low-technology equipment such as Visual Inspection with Acetic Acid (VIA) for screening and cryotherapy ablative treatment of VIA-positive precancerous lesions [9].

In 2004, Malawi adopted the World Health Organization (WHO) endorsed screen-and-treat VIA-based cervical cancer control program. By 2011, the program was scaled up to all central and district hospitals. Evaluation of the program between 2011 and 2015 revealed high failure rate of treating VIA-positive lesions with cryotherapy: only 43% of the 2311 women eligible were actually treated [10]. The difficulty in sustaining the treatment component of the initiative was largely due to high running cost of cryotherapy, such as securing refrigerant gas [10]. Another major barrier to uptake is the long travel distance to health facilities, hindering women’s access to services [11, 12]. Further barriers identified are low knowledge, community stigma and misconception of cervical cancer and cervical cancer screening [13, 14].

To overcome the barriers stated above, we implemented a pilot project in rural Malawi in 2017 to bring screen-and-treat utilizing VIA and thermocoagulation directly to the community [15]. Thermocoagulation is an alternative ablative therapy that can use battery, is more portable than cryotherapy, with efficacy and safety comparable to cryotherapy [16–18]. It has been successfully utilized for screening programs in Malawi with high cure rates (93.3% at 6 months, comparable to cryotherapy [18]) and as of 2019, it has been recommended by WHO as treatment for precancerous lesions in LMIC [19]. However its use in the field, outside health facilities, has not been well studied. Therefore, we implemented a community-based pilot program utilizing VIA and same day thermocoagulation for cervical cancer screening. This qualitative sub-study was implemented to assessed participants’ experience and acceptability of this approach, as well as attitudes towards alternative community-based screening strategies such as HPV self-sampling.

## Methods

### Study setting and participants

A community-based cervical cancer screen-and-treat pilot program with VIA and thermocoagulation was implemented by UNC Project-Malawi between July–August 2017 in 4 villages in rural Lilongwe, Malawi. The recruitment and screening protocols are detailed elsewhere [15]. In brief, we first met with the local traditional leaders to discuss the screening program. Once we received permission to proceed, we conducted educational talks about cervical cancer through community meetings on days leading up to the screening. On the screening day for each village, we arrived with our equipment and set up in predesignated areas. The study population comprised of women between 25 and 50 years of age who had never been diagnosed with cervical cancer [15]. We offered participants VIA screening and same-day thermocoagulation treatment for VIA positive lesions eligible for ablative therapy. Women who received thermocoagulation were followed-up at 6 and 12 weeks with UNC-Project Malawi study team located at Kamuzu Central Hospital (KCH) in Lilongwe. Participants were offered participation in an exploratory qualitative sub-study at their 12-week follow-up using convenience sampling. We aimed to interview 10 participants who attended their 12-week follow-up visit and 10 participants who missed their follow-up to capture differences in experiences and barriers to care. Those who did not attend the 12-week visit were traced by study staff and their visits rescheduled. Participants were required to present follow-up appointments at KCH on their own, however reimbursement for transportation was provided upon arrival.

### Data collection

We used 7 domains of inquiry to guide semi-structured interviews: 1) baseline knowledge of cervical cancer; 2) perception of cervical cancer screening; 3) screen-and-treat experience; 4) acceptability of the pilot screening program; 5) follow-up challenges; 6) community and partner support; 7) attitudes towards self-sampling for HPV testing as alternative screening method. Domains of inquiry were developed based on existing literature exploring barriers to cervical cancer screening in SSA [13, 14, 20–23] and assessment of screening programs in in LMIC [24–28]. The interviews were conducted in the local language, Chichewa, by study staff experienced in qualitative data collection methods (MT). The interviews were audiotaped, then translated and simultaneously transcribed to English.

### Data analysis

A codebook was generated during the coding process through agreement among the 3 coders (FL, AB, JC). Content analysis was performed using NVIVO v12 and in-code memos between transcriptions were used to assure reliability of coding and validity of findings. In this paper, we present the results of domains 3, 4 and 7 of the qualitative sub-study.

### Ethical considerations

This study was approved by both the Malawi National Health Sciences Research Committee and the University of North Carolina at Chapel Hill Institution Review Board. All participants provided written informed consent after completing the informed consent process.

## Results

Between July–August 2017, 408 women were screened with VIA and 30 were VIA positive, of whom 28 underwent same day thermocoagulation. Ten of the 28 participants failed to present to their scheduled 12-week follow-up visits, of whom 7 were successfully traced and accepted the interview. We therefore interviewed a total of 17 women who received thermocoagulation: 10 participants who presented for their follow-up visit and 7 who did not and required tracing. We found that themes were well saturated after the 17 interviews.

I. Baseline characteristics of the women in the qualitative sub-study

Of the 17 women interviewed, almost all were between 30 and 40 years of age, only 2 reported some secondary education, 15 were married, and 4 were in a polygamous relationship; however, 11 reported that their partners had other partners (Table 1). Notably, no participant interviewed were HIV positive.

II. Participants had an overall positive screen-and-treat experience

**Table 1:** Baseline characteristics of study participants

Participant Demographics	(***N*** = 17)
**Age**	**% (n)**
20–29	12% (2)
30–39	47% (8)
40–49	35% (6)
50–59	6% (1)
**Level of education**	
No formal	24% (4)
Primary school	70% (12)
Secondary school	6% (1)
**Marital status**	
Married monogamous	65% (11)
Married polygamous	24% (4)
other	12% (2)
**Total lifetime partners**	
1 partner	35% (6)
2–3 partners	59% (10)
> 4 partners	6% (1)

Participants’ experience of the entire screen-and-treat process was explored, including how the procedure was tolerated, any concerns throughout the process, how well they understood the screening and their final perception of program. All 17 women reported an overall positive experience. One woman reported:

*“For me, I felt that it was a very precious thing to know and be visited by doctors in our village.”* (ID#269, age 36).

Her statement reflects a common theme of appreciation for community-based screening among many participants. Other themes included feeling that they were well counseled and knew what to expect through each step of the screen-and-treat process. For example, this participant expressed appreciation for anticipatory guidance and counseling:

*“They explained to us everything that was going to happen the screening procedure, so we knew that it’s either we will be found with cancer or not. They also explained to us that if you have been found with the cancer cells, they have equipment which they use for thermocoagulation, they explained everything beforehand.”* (ID#385^1^, age 39).

Many women also reported appreciation for immediate treatment, which provided relief and decreased anxiety surrounding a positive VIA screen:

“*I was comfortable because before everything, they told us that whoever they found with the cancer cells, they will burn them right away.”* (ID#293, age 36).

Most reported tolerating the thermocoagulation very well. One participant reflected on her experience in detail, demonstrating that thermocoagulation was painless and well tolerated:

*“[The doctor] showed me an equipment saying, ‘This is the instrument that we are going to use…do you agree that we should burn the abnormal cells?’ I said I agreed, and he said ‘okay’ …He burnt for the first time and asked me ‘Do you feel pain?’ I said, ‘I am not feeling any pain.’”* (ID#171, age 42).

III. Negative experiences during screening were rare

Negative experiences were rare and minor. One participant reported discomfort during the procedure that appeared to be consistent with discomfort of a metal speculum exam and not of thermocoagulation itself:

*“They were inserting metals inside us, it being the first time, it was a bit difficult and I experienced some pain.”* (ID#269, age 26).

Another woman noted that she had pain only during the procedure, which resolved immediately after:

*“I would close my legs even during the procedure, you know, it was painful [laughs]… from the time I stood up and went home I did not have any difficulties or any other pain”* (ID#329^1^, age 41).

Other participants reported post-procedural vaginal discharge, which resolved shortly after, but the majority felt well counseled on what to expect and was not worried, as described by this participant:

*“After the screening was done, they told me that I would experience excess vaginal discharge for 2 weeks and indeed that was what happened… after 2 weeks I noticed that the vaginal discharge stopped and since that time, I haven’t experienced any kind of pain”* (ID#329^1^, age 41).

Finally, one participant noted that the long duration of screening was challenge for her, especially when her exam required a second opinion as described here:

“*The most difficult part of the entire screening process to me was when the doctor explained to me that it was not clear to her whether I had the cancer cells or not, and she called another one in. That was when I had difficulties because it took more time.”* (ID#171, age 42).

IV. Participants found it difficult to maintain postprocedural abstinence due to male partners

Many participants felt obligated to tell their male partners that they underwent screening and received treatment for precancerous lesions due to the recommendation of post-procedural abstinence for 6 weeks to allow for the cervix to heal after thermocoagulation. Some reported that while their husbands were supportive of their decision to proceed to screening and initially accepted the abstinence recommendation, participants ultimately experienced pressure from their male partners and were unable to comply. For example, one participant reported:

*“After I told him [about the screening], he did not understand…We managed to stay for 2 weeks without sex but that was because he was angry. The third week however things got worse and then we had sex. So, it only worked for 2 weeks and that was because he was angry.” (ID329*^1^*, age 41)*.

For those who were able to follow the recommendations of post-procedural abstinence for the entire 6 weeks, several reported that it was likely their male partners had other partners during that time. This participant reported that her husband took another partner during this time, but ultimately expressed gratitude for accessing screening and treatment:

*“After I told him that we should stay 6 weeks without sex, I think he found it difficult not to have sex for 6 weeks. In the end, he got another woman and started to stay with her. I asked myself… is 6 weeks such a long time that he must take another woman? Then how did he manage to wait without having sex after I had given birth? I then made up my mind and decided that all that should not bother me, so long as at the end of it all I get cured from cancer.”* (ID#207^1^, age 32)

Given the challenges expressed by these participants, all felt that male partners should be included in the counseling surrounding cervical cancer screening so that they can be more supportive in post-procedural needs such as abstaining from sex or presenting to follow-ups if needed. It was also echoed among many participants that healthcare workers should be responsible in educating males in the community about cervical cancer.

*“You [healthcare workers] are the ones to encourage men to have counselling sessions together with us. If they [male partners] understand, they would be able to encourage their wives to go for screening. You can explain to them because most of them have women in their homes so they should be able to understand the dangers of not being screened. No man would want his wife to die and leave him with kids”* (ID#269, age 26)

Most women felt that that male partner should play a larger role in cervical cancer prevention in the way of understanding, encouragement and providing transportation money to health facilities if needed.

V. Participants demonstrated increased understanding of cervical cancer prevention after the screen-and-treat experience

All participants were able to recount the screening process, some in more detail than others, even over 12 weeks after screening. Most described a positive VIA screen as finding “cancer cells,” while others described it as detecting “cells,” “germs” or “virus.”

*“Telling me that they have found some cancer cells inside my cervix, I felt that it was very important and worth following the advice given to me.*” (ID#269, age 26)

It was clear throughout the interviews that participants understood the purpose of screening for early detection and treatment to prevent worsening disease.

*“I have learnt that cervical cancer is real and that we should not ignore calls by the hospital to go for screening, because if detected early, then we can get help right away.”* (ID#239, age 36)

While the understanding of screening for “precancer” was not always distinguished from “cancer,” the concept of treatment for prevention of worsening disease resonated with most participants. It was also evident in all interviews that the understanding of cervical cancer and prevention improved after participating in the screening process. This woman described the knowledge she gained and the desire to share it with others, demonstrating the effectiveness of the counseling:

*“So, my understanding was that the abnormal VIA screening result on the cervix is the beginning of cancer. So, in the community I was not hiding, I was able to share the results with other women and encouraging them to do the same, because if it’s been detected early, you will be able to get treatment.”* (ID#240, age 36)

Her desire to share her experience with other women was echoed by several others.

Women also noted that the campaign dispersed some of their initial fears and misconceptions about getting screened:

*“Even though we went for the screening, it was just out of bravery and the need to be assisted. After the screening, we realized that we were simply scaring each other, there is nothing scary! Because like I have said, when you are diagnosed when the disease has already become worse, it is often difficult to get better. That is also why they say prevention is better than cure.”* (ID#385^1^, age 39)

For some participants, the experience was so positive that they wanted to make sure other women would also benefit:

*“The campaign should be intensified and reach more women all over the country so that more women can benefit from the service. I will use my story and experience as a tool to encourage my fellow women to go for cervical cancer screening, because I am a living example.”* (ID#240, age 36)

VI. *Self-sampling for HPV testing may be an acceptable alternative for community-based screening*

At the end of the interview, participants were asked about how they, or women in their community, would feel about self-collection of samples for cervical cancer screening for HPV testing, as an alternative to screening with VIA. Interviewers explained to participants that “it involves women taking their own vaginal fluid using a cotton swab from the vagina and handing it to the clinicians or the hospital.” It was further clarified that unlike VIA, HPV results may not be immediately available, could take several hours or days, and women would potentially have to present for treatment at a later time.

In general, participants reported that this could be an acceptable method for those who are hesitant about speculum exams:

*“For some people who may fear the instruments, they may use this method (self- sampling) to collect the swab themselves.”* (ID#180^1^, age 31).

Or for those constrained by fear or community stigma of visiting a healthcare facility:

*“This method [self-sampling] could work well with those with difficult husbands, so maybe those women can follow this method. Those women who are also shy could benefit from this method.”* (ID#385^1^, age 39)

Concerns expressed by participants were not being able to collect it correctly, having to travel to the clinic to receive results, long wait time for results and not receiving treatment immediately following a positive result. Others saw value in in-person evaluation from a healthcare professional:

*“It is difficult that once you deliver the self-sample you are gone, unlike when the doctors perform the [pelvic exam and VIA screening], because they will give you treatment. Waiting for the results is difficulty to bear...the treatment you get right after screening is different from one you will get a day after”* (ID#269, age 26)

These concerns resonated with prior themes of valuing immediate result and same-day treatment as well as community-based screening to avoid travel challenges. However, overall, participants expressed that self-collection could potentially be an acceptable alternative to VIA screening in their community.

## Discussion

Our community-based screen-and-treat with VIA and thermocoagulation was well tolerated and accepted by the women interviewed in this study. They valued being able to participate in screening and treatment in their own community. This highlights the success of the pilot program to overcome access limitations associated with distance. However, travel to health facilities for follow-up appears to remain a significant barrier for our participants, as evident by the 10 participants in the pilot study that found it difficult to present for the study follow-up visit despite the provision of transport reimbursement through the study.

### Same-day treatment is important in cervical cancer prevention

The participants interviewed valued same-day treatment of VIA lesions. Loss to follow-up is not uncommon in cervical cancer screening programs in sub-Saharan Africa. A study from Zambia showed that 22% of women that chose to defer cryotherapy did not return for treatment [29]. Similarly, over 500 of the 2311 women with cervical lesions requiring ablative therapy in the Malawi national cervical cancer screening program never received treatment when cryotherapy was postponed [10]. Fear of a positive screen has been reported as a barrier to screening in many SSA communities [30, 31], and immediate treatment also alleviated anxiety surrounding a positive screen. This study reinforces that same-day treatment is critical in successful cervical cancer prevention as it can decrease loss to follow-up and provide a peace-of-mind for participating women.

### Thorough counseling is important in understanding cervical cancer screening

Another major theme echoed by many participants was the appreciation for counseling. Women felt that they received adequate anticipatory guidance during each step of the screening process, allowing them to understand the process and what to expect. The pilot study implemented community education prior to enrollment and continued to counsel patients throughout. Women were able to describe the screen-and-treat process in detail, even over 12 weeks after their experience. They demonstrated understanding of early detection and treatment for prevention of worsening disease. They showed increased knowledge of cervical cancer and prevention strategies. Many even felt they wanted to share their experiences with other women to help clarify misconceptions that keep women from presenting for screening. Other studies that have evaluated women’s screening experience have also found that cohort knowledge improved after undergoing screening due to the counseling that was provided [26]. This finding could, however, also be due to self-selection of women who participated in screening also having more motivation to seek information. Nevertheless, our findings demonstrate that understanding the screening process and purpose of early treatment are fundamental to women feeling empowered and engaging in preventative health.

### Male partners should be included cervical cancer prevention strategies

The major concern expressed by participants was the difficulty in following the recommended 6-week post-procedural abstinence. Some felt they had to tell their male partners about the treatment to negotiate abstinence, and many were unable to comply due to pressure from their male partners. Participants further expressed desire for male partner involvement in cervical cancer screening. Many felt that healthcare providers should be providing education to their partners. Male partners have been identified as barrier to screening in studies across SSA [13, 32–34], but also as source of support to encourage screening and follow-up [35, 36]. Male partners are an untapped support system in the community to facilitate cervical cancer prevention and all cervical cancer prevention strategies should include and prioritize a male partner education and awareness component.

### Self-sampling HPV screening may be an acceptable alternative for screening in this community

Due to the high sensitivity and reproducibility of HPV DNA testing in both HIV-infected and uninfected women, WHO now recommends HPV screen-and-treat as strategy for cervical cancer control in LMICs when available [35, 37, 38]. HPV testing can be performed on self-collected sample, removing the need for a clinic visit, trained providers and pelvic exams. A study in Argentina showed a 4-fold increase in uptake of cervical cancer screening with the addition of self-collection of samples for HPV testing by community health workers during home visits [39]. Studies in SSA on HPV self-sampling have also shown high acceptability in Kenya and Uganda [27, 40, 41]. While the strategy of HPV self-sampling can facilitate community-based screening, decrease the resources needed and perhaps appeal to more women, the success of this strategy depends on its acceptability among women in each target community.

In our study, there was agreement that self-sampling appeared to be an acceptable alternative method to cervical cancer screening for certain women in the community, but most participants preferred the screening they had received. The main concerns for HPV self-sampling in our study were consistent with findings from a quantitative study that evaluated 824 Malawian women’s willingness to self-collect samples for HPV in Lilongwe, Malawi. Though 67% of the women reported willingness to self-collect a vaginal sample, reported concerns were that it might hurt (22%), might not be collected correctly (21%), might not be accurate (17%). In addition, 5% of the women reported that they would rather go to the health facility [28]. These concerns can help inform counseling strategies, specifically regarding adequacy of sampling, when the results will be available and how to access treatment in a timely manner if positive result.

### Study limitations

Selection bias is a limitation of this study. Our interviews selectively demonstrated the views of women in Lilongwe who completed our community-based screening program, including VIA and same-day thermocoagulation. It does not capture the perspectives of those who did not participate, those who did not receive thermocoagulation or those who were lost to follow-up. Non-screening participants could provide alternative insight into screening preferences and self-collection perspectives important to expanding screening in this community. We were also unable to interview 3 of the women who did not return for their 12-week visit, these women may have had more negative experiences than was captured in this analysis. In addition, the interviews were conducted at least 12 weeks after thermocoagulation, therefore participants were vulnerable to recall bias. While recall bias could be potentially mitigated by conducting part of the interview on the same day as the screening, the concern is that the addition of interviews could result in a longer day for participants and lead to research interruptions to the experience. Finally, participants may have felt more obligated to report more positive experiences when interviewed in a research setting. This can be mitigated perhaps by conducting interviews in participants’ homes or communities as appropriate.

## Conclusion

Innovative cervical cancer screening strategies that meet the needs of the community and overcome barriers are essential in reducing the global burden of cervical cancer. Effective implementation of cervical cancer prevention services requires reaching the target population with programs that are acceptable, sustainable and raise awareness for the entire community. This study evaluated in-depth the experiences of participants who completed a screening program that utilized same-day thermocoagulation to treat VIA-positive participants in their communities. Our findings demonstrate that the procedures were well-tolerated, the strategy was acceptable and furthermore, highlighted aspects of screening the participants valued most: same-day treatment for VIA-positive, thorough counseling and anticipatory guidance on what to expect with screening and treatment, recommendations to include male partners in prevention strategies and that self-sampling HPV testing may be an acceptable way to increase screening access for other women in the community. While only a small cohort of 17 participants were interviewed, the themes were well saturated. While findings from qualitative studies are not meant to be generalized, the depth of information we gathered is insightful and can be used to further tailor screening strategies to this community and other similar settings. On-going investigations for scale-up of screening at a national level include gaining perspectives of women other communities (as priorities and acceptability may differ), incorporating strategies to reach more rural communities and increase education of male partners. Future consideration will be to include self-sampling HPV as alternative primary screening method to allow for more privacy, reduce need for pelvic exams and reach more women in the community.

## Supplementary Information

**Additional file 1.**

**Additional file 2.**

## Data Availability

Data available from corresponding author on request.

## Abbreviations

HIV: Human Immunodeficiency Virus
HPV: Human Papilloma Virus
VIA: Visual Inspection with acetic acid
SSA: Sub-Saharan Africa
LMIC: Low and middle-income countries
WHO: World Health Organization
UNC: University of North Carolina
KCH: Kamuzu Central Hospital

## Footnotes

1 Missed initial follow-up visit
